# Small RNA profiling and degradome analysis reveal regulation of microRNA in peanut embryogenesis and early pod development

**DOI:** 10.1186/s12864-017-3587-8

**Published:** 2017-03-02

**Authors:** Chao Gao, Pengfei Wang, Shuzhen Zhao, Chuanzhi Zhao, Han Xia, Lei Hou, Zheng Ju, Ye Zhang, Changsheng Li, Xingjun Wang

**Affiliations:** 1 Biotechnology Research Center, Shandong Academy of Agricultural Sciences, Shandong Provincial Key Laboratory of Crop Genetic Improvement, Ecology and Physiology, Jinan, 250100 People’s Republic of China; 2 College of Life Sciences, Shandong Normal University, Jinan, 250014 People’s Republic of China; 3 Department of Food Biotechnology, College of Food Science and Nutritional Engineering, China Agricultural University, Beijing, 100083 People’s Republic of China

**Keywords:** High-throughput sequencing, Peanut, miRNA, Hormone, Light, Embryogenesis, Pod development

## Abstract

**Background:**

As a typical geocarpic plant, peanut embryogenesis and pod development are complex processes involving many gene regulatory pathways and controlled by appropriate hormone level. MicroRNAs (miRNAs) are small non-coding RNAs that play indispensable roles in post-transcriptional gene regulation. Recently, identification and characterization of peanut miRNAs has been described. However, whether miRNAs participate in the regulation of peanut embryogenesis and pod development has yet to be explored.

**Results:**

In this study, small RNA and degradome libraries from peanut early pod of different developmental stages were constructed and sequenced. A total of 70 known and 24 novel miRNA families were discovered. Among them, 16 miRNA families were legume-specific and 12 families were peanut-specific. 30 known and 10 novel miRNA families were differentially expressed during pod development. In addition, 115 target genes were identified for 47 miRNA families by degradome sequencing. Several new targets that might be specific to peanut were found and further validated by RNA ligase-mediated rapid amplification of 5′ cDNA ends (RLM 5′-RACE). Furthermore, we performed profiling analysis of intact and total transcripts of several target genes, demonstrating that *SPL* (miR156/157), *NAC* (miR164), *PPRP* (miR167 and miR1088), *AP2* (miR172) and *GRF* (miR396) are actively modulated during early pod development, respectively.

**Conclusions:**

Large numbers of miRNAs and their related target genes were identified through deep sequencing. These findings provided new information on miRNA-mediated regulatory pathways in peanut pod, which will contribute to the comprehensive understanding of the molecular mechanisms that governing peanut embryo and early pod development.

**Electronic supplementary material:**

The online version of this article (doi:10.1186/s12864-017-3587-8) contains supplementary material, which is available to authorized users.

## Background

Peanut (*Arachis hypogaea* L.) is an important crop grown world widely for both oil and protein production. The development of peanut embryo is inhibited by light above ground, and the development of embryo and pod resumes after the elongated ovaries are buried into soil [1–3]. This special developmental process of peanut fruit is a complex, genetically programmed process involving many gene regulatory networks at the transcriptional and post-transcriptional levels. Dissecting the molecular mechanism governing peanut embryo and early pod development is helpful to broaden our knowledge on plant embryogenesis. Previous studies demonstrated that peanut embryogenesis and pod development were affected by different wavelengths of light. For example, continuous irradiation with white, red or blue light inhibited embryogenesis and pod development whereas darkness or far red light promoted this process [4–7]. Gynophore elongation responded to light in the opposite manner, which was stimulated when grown in white, red or blue and inhibited when grown in darkness or far red light [6]. Besides, plant endogenous hormones such as auxin (IAA), gibberellic acid (GA), ethylene, abscisic acid (ABA) and brassinolides (BRs) are well known to play critical roles in embryo and fruit development [8, 9]. In peanut, it was reported that either the content or the distribution patterns of hormones significantly changed during peanut early pod development [10–12]. It has been shown that low concentration of IAA promotes peanut pod development, whereas high level inhibits peanut gynophore elongation [13, 14]. GAs can also promote the growth of gynophores in peanut. However, how light regulates hormone biosynthesis and signaling to initiate this interesting biological process is unknown.

MicroRNAs (miRNAs) are a class of small non-coding RNAs with approximately 20–22 nt in length. MiRNAs regulate gene expression at the post-transcriptional level in almost all eukaryotes [15]. In general, miRNAs specifically target messenger RNAs (mRNAs) to inhibit their translation or induce their cleavage through partially or fully sequence complementary with their targets [16, 17]. The past decade has witnessed an explosion in our knowledge on miRNA regulation in various biological processes in plants. MiR156 and miR172 coordinately regulate the timing of juvenile-to-adult transition during shoot development [18]. Overexpression of miR167 in wild tomato causes a defect in flower development and female sterility through suppressing *Auxin Response Factor 6* (*ARF6*) and *Auxin Response Factor 8* (*ARF8*) [19]. Both miR156 and miR397 are involved in the regulation of seed development by controlling grain size and shape in rice [20, 21]. Increasing evidence indicated that miRNA and hormone signaling interact to regulate those physiological processes. For examples, GA was shown to modulate miR159 levels during Arabidopsis seed germination [22]. However, our knowledge on miRNA functions controlling the species-specific biological processes in plants is quite limited.

Our previous report has identified miRNAs from peanut root, leaf and stem using deep sequencing approach [23]. However, there is no report on miRNA regulation in peanut embryogenesis and early pod development and no functional miRNA-mRNA modules have been identified from peanut pod. To gain a better understanding of the function of miRNA in peanut embryogenesis and early pod development, the current study characterized the expression profiles of miRNAs in gynophores of three developmental stages during which the repressed embryo and ovary reactivate for further development. Additionally, the degradome library sequencing for global identification of miRNA targets in peanut was performed and new target genes were discovered., many of which involved in plant hormone signal transduction processes. These findings hinted at the important roles of miRNAs in regulating peanut embryogenesis and early pod development and constructed an outline for the interaction between light signal, hormone and miRNAs during peanut embryo and early pod development.

## Results

### Overview of small RNA profiles in peanut gynophores

**Fig. 1:**
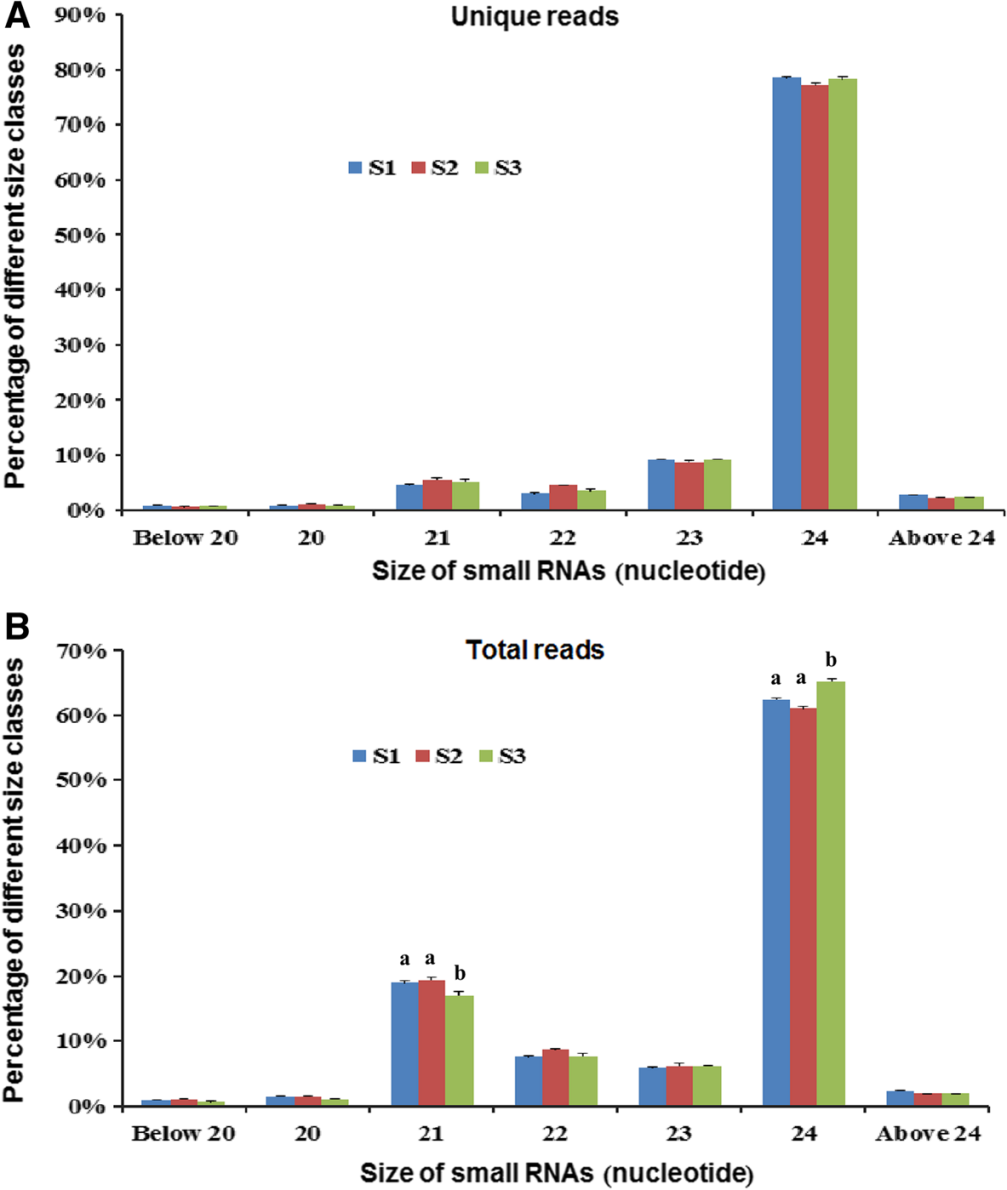
Length distribution of small RNA reads in S1, S2 and S3 gynophores. **a** The distribution of unique small RNAs that present in two biological replicates. **b** The distribution of total small RNAs that present in two biological replicates

To assess the regulatory roles of miRNAs in peanut embryogenesis and early pod development, we profiled sRNA accumulation in S1, S2 and S3 gynophores (Fig. 3a). More than 12 million total reads and 6 million unique reads (stand for read species) were produced from each sample. About 78% of the total reads and 81% of the unique reads were perfectly mapped to peanut genome, and the rates of genomic match were similar across these three stages (Additional file 1: Table S1). The correlation coefficients were more than 0.97 between two biological replicates (Additional file 2: Figure S1). As shown in Fig. 1, 24 nt class of sRNAs showed the highest abundance (~60% of the total and 78% of the unique reads). The secondly abundant class of total reads was 21 nt sRNAs (~19%). This result was consistent with that found in rice [24], tomato [25], soybean [26] and a previous study in peanut [23], but different from that of wheat and grapevine [27, 28]. The proportion of unique reads has no obvious difference among three stages. Interestingly, the proportion of 21 nt total reads decreased slightly and the proportion of 24 nt total reads increased at S3 compared with S1 and S2 (Fig. 1). The size distribution of 20, 22 and 23 nt total reads has no obvious difference among three stages. After removal of rRNA, tRNA, snRNA, snoRNA, repeats sequence and exon sequence (for statistics on read counts, see Additional file 1: Table S1), the remaining unique reads that present in two biological replicates were used to identify miRNAs subsequently.

### Identification of known and novel miRNAs in peanut gynophore

To identify known miRNAs in peanut, all the unannotated unique reads that perfectly mapped to peanut genome were aligned to plant miRNAs in miRBase (Release 21.0, June 2014). A total of 104 known miRNAs belonging to 70 families were identified (Table 1). Among them, 39 families were known and well conserved that present in two or more plant species. In addition, 19 families were also known but less conserved that present only in one plant species including miR894, miR1088, miR1520, miR2199 and others. Furthermore, 12 peanut-specific miRNA families loaded in miRBase were also detected in our study, for example, miR3508, miR3509, miR3511, and miR3512. After identification of known miRNAs, the remaining unique reads were used to identify novel miRNAs by predicting the hairpin structures of their precursor sequences. 27 novel miRNAs belonging to 24 families were identified in this study and were named as miRn1 to miR24 (Additional file 3: Table S3). The corresponding miRNA* sequences of 15 novel miRNAs were detected, further supporting the existence of these miRNAs. Most novel miRNAs could only be produced from one locus, except miRn10 and miRn23, which were produced from three and four loci, respectively (Additional file 3: Table S3). Stem-loop RT-PCR was performed to validate the predicted new miRNAs and 15 predicted miRNAs were found to be expressed in peanut gynophore (Additional file 4: Figure S2).

**Table 1:** Known and novel miRNA families identified in peanut gynorphore

Well-conserved	Mature sequence	Length(nt)	Star(*)	References	Conserved in other plants
miR156	UGACAGAAGAGAGUGAGCAC	20	Yes	Zhao et al., 2010 [24]; Chi et al., 2011 [35].	Arabidopsis, Rice, Maize et al.
	UUGACAGAAGAGAGUGAGCAC	21	Yes		
	UGAUAGAAGAGAGUGAGCACA	21	Yes		
	UUGACAGAAGAGAGUGAGCACA	22	Yes		
miR157	UUGACAGAAGAUAGAGAGCAC	21	Yes	Zhao et al., 2010 [24]; Chi et al., 2011 [35].	Arabidopsis, Rice, Maize et al.
	UGACAGAAGAUAGAGAGCACA	22	Yes		
	UUGACAGAAGAUAGAGAGCA	20	Yes		
miR159	UUUGGAUUGAAGGGAGCUCUA	21	Yes	Zhao et al., 2010 [24]; Chi et al., 2011 [35].	Arabidopsis, Rice, Maize et al.
	UUUGGAUUGAAGGGAGCUCU	20	Yes		
miR160	UGCCUGGCUCCCUGUAUGCCA	21	Yes	Zhao et al., 2010 [24]; Chi et al., 2011 [35].	Arabidopsis, Rice, Maize et al.
miR162	UCGAUAAACCUCUGCAUCCAG	21	Yes	Zhao et al., 2010 [24]; Chi et al., 2011 [35].	Arabidopsis, Rice, Maize et al.
miR164	UGGAGAAGCAGGGCACGUGCA	21	No	Zhao et al., 2010 [24]; Chi et al., 2011 [35].	Arabidopsis, Rice, Maize et al.
	UGGAGAAGCAGGGCACGUGC	20	No		
	UGGAGAAGCAGGGCACGUGCAA	22	No		
	UGGAGAAGCAGGGCACGUGCAAU	23	No		
miR165	UCGGACCAGGCUUCAUUCCUC	21	Yes	Zhao et al., 2010 [24]; Chi et al., 2011 [35].	Arabidopsis, Rice, Maize et al.
	UCGGACCAGGCUUCAUUCC	19	Yes		
miR166	UCGGACCAGGCUUCAUUCCCC	21	Yes	Zhao et al., 2010 [24]; Chi et al., 2011 [35].	Arabidopsis, Rice, Maize et al.
	UCUCGGACCAGGCUUCAUUCC	21	Yes		
	UCGGACCAGGCUUCAUUCCC	20	Yes		
	UCGGACCAGGCUUCAUUCC	19	Yes		
miR167	UGAAGCUGCCAGCAUGAUCUU	21	Yes	Zhao et al., 2010 [24]; Chi et al., 2011 [35].	Arabidopsis, Rice, Maize et al.
	UGAAGCUGCCAGCAUGAUCU	20	Yes		
	UGAAGCUGCCAGCAUGAUCUUA	22	Yes		
miR168	UCGCUUGGUGCAGGUCGGGAA	21	Yes	Zhao et al., 2010 [24]; Chi et al., 2011 [35].	Arabidopsis, Rice, Maize et al.
	UCGCUUGGUGCAGGUCGGGA	20	Yes		
	CGCUUGGUGCAGGUCGGGAAC	21	Yes		
miR169a	AAGCCAAGGAUGACUUGCCGG	21	Yes	Zhao et al., 2010 [24]; Chi et al., 2011 [35].	Arabidopsis, Rice, Maize et al.
miR169b	GGCAGGUCAUCUUGUGGCUAU	21	Yes		
	GGCAGGUCAUCUUGUGGCUAUA	22	Yes		
miR171	GGAUAUUGGUGCGGUUCAAUG	21	Yes	Zhao et al., 2010 [24]; Chi et al., 2011 [35].	Arabidopsis, Rice, Maize et al.
	UAUUGGUGCGGUUCAAUGAGA	21	Yes		
miR172	AGAAUCUUGAUGAUGCUGCAU	21	Yes	Zhao et al., 2010 [24]; Chi et al., 2011 [35].	Arabidopsis, Rice, Maize et al.
	AGAAUCUUGAUGAUGCUGCA	20	Yes		
miR319	UUGGACUGAAGGGAGCUCCCU	21	Yes		Arabidopsis, Rice, Maize et al.
miR390	AAGCUCAGGAGGGAUAGCGCC	21	Yes	Zhao et al., 2010 [24]; Chi et al., 2011 [35].	Arabidopsis, Rice, Maize et al.
miR391	UGUCGCAGGAGAAAUAGCACC	21	No		Arabidopsis, Rice, Maize et al.
miR393	UCCAAAGGGAUCGCAUUGAUC	21	Yes	Zhao et al., 2010 [24]; Chi et al., 2011 [35].	Arabidopsis, Rice, Maize et al.
miR394	UUGGCAUUCUGUCCACCUCC	20	Yes	Zhao et al., 2010 [24]; Chi et al., 2011 [35].	Arabidopsis, Rice, Maize et al.
miR396	UUCCACAGCUUUCUUGAACUU	21	Yes	Zhao et al., 2010 [24]; Chi et al., 2011 [35].	Arabidopsis, Rice, Maize et al.
	GCUCAAGAAAGCUGUGGGAGA	21	Yes		
	CUCAAGAAAGCUGUGGGAGA	20	Yes		
miR397	UCAUUGAGUGCAGCGUUGAUG	21	Yes	Zhao et al., 2010 [24]; Chi et al., 2011 [35].	Arabidopsis, Rice, Maize et al.
miR398	UGUGUUCUCAGGUCACCCCUU	21	Yes	Zhao et al., 2010 [24]; Chi et al., 2011 [35].	Arabidopsis, Rice, Maize et al.
miR399	GGGCACCUCUUCACUGGCAUG	21	Yes	Zhao et al., 2010 [24]; Chi et al., 2011 [35].	Arabidopsis, Rice, Maize et al.
miR403	UUAGAUUCACGCACAAACUUG	21	Yes	Chi et al., 2011 [35].	Arabidopsis, Soybean et al.
miR408	CUGGGAACAGGCAGAGCAUGA	21	No	Zhao et al., 2010 [24]; Chi et al., 2011 [35].	Soybean, Maize et al.
miR414	AACAGAGCAGAACAGAACAGA	21	No		Arabidopsis, Rice et al.
miR477	UCCCUCAAAGGCUUCCAGUA	20	No		Physcomitrella patens, Grape et al.
	UCCCUCAAAGGCUUCCAGUAU	21	No		
miR482a	GGAAUGGGCGGUUUGGGAUGA	21	Yes		Arabidopsis, Rice, Maize et al.
miR482b	UUCCCAAUUCCACCCAUUCCUA	22	Yes		Arabidopsis, Rice, Maize et al.
miR530	UGCAUUUGCACCUGCACUUUA	21	No		Arabidopsis, Soybean, Alfalfa et al.
miR845	CCAAGCUCUGAUACCAAUUGAUGG	24	No		Arabidopsis, Grape et al.
miR1507	CCUCGUUCCAUACAUCAUCUAA	22	Yes	Chi et al., 2011 [35].	Soybean, Alfalfa
	CCCUCGUUCCAUACAUCAUCUA	22	Yes		
miR1509	UUAAUCAAGGGAAUCACAGUUG	22	No		Soybean, Alfalfa
	UUAAUCAAGGGAAUCACAGUU	21	No		
miR1511	AACCAGGCUCUGAUACCAUGA	21	No	Chi et al., 2011 [35].	Soybean, Alfalfa
miR1515	UCAUUUUUGCAUGCAAUGAUCC	22	No	Chi et al., 2011 [35].	Soybean, Alfalfa
miR2111	AUCCUUAGGAUGCAGAUUACG	21	No	Chi et al., 2011 [35].	Soybean, Alfalfa
miR2118	UUGCCGAUUCCACCCAUGCCUA	22	No	Chi et al., 2011 [35].	Soybean, Alfalfa
	UUGCCGAUUCCACCCAUGCCU	21	No		
miR4376	ACGCAGGAGAGAUGGCGCUAU	21	No		Soybean, Tomato et al.
	UACGCAGGAGAGAUGGCGCUA	21	No		
miR4414	AGCUGCUGACUCGUCGGUUCA	21	Yes		Soybean, Alfalfa
	AGCUGCUGACUCGUCGGUUC	20	Yes		
miR5225	UCUGUCGCAGGAGAGAUGACG	21	No		Arabidopsis, Soybean, Alfalfa et al.
	UCUGUCGCAGGAGAGAUGACGC	22	No		
Less-conserved	Mature sequence	Length(nt)	Star(*)	References	Conservative in other plants
miR829	AAGCUCUGAUACCAAUUGAUGGUU	24	No		Arabidopsis
miR894	CGUUUCACGUCGGGUUCACCA	20	No	Zhao et al., 2010 [24]; Chi et al., 2011 [35].	Physcomitrella patens
miR1088	UGACGGAAGAAAGAGAGCACA	21	Yes		Physcomitrella patens
	UUGACGGAAGAAAGAGAGCAC	21	Yes		
	UUGACGGAAGAAAGAGAGCACA	22	Yes		
miR1520	AUGUUGUUAAUUGGAGGAGCGG	22	No		Soybean
	UGUUGUUAAUUGGAGGAGCGGU	22	No		
miR2084	CGUCAUCGUUGCGAUUGUGGA	21	No		Physcomitrella patens
miR2199	UGAUACACUAGCACGGGUCAC	21	No	Chi et al., 2011 [35].	Alfalfa
miR2628	GAAGAAAGAGAAUGAUGAGUAA	22	No		Alfalfa
miR5021	UGAGAAGAAGAAGAAGAAGAA	21	No		Arabidopsis
miR5221	AGGAGAGAUGGUGUUUUGACUU	22	No		Alfalfa
miR5227	UGAAGAGAAGGGAUUUAUGAA	21	No		Alfalfa
miR5234	UGUUAUUGUGGAUGGCAGAAG	21	No		Alfalfa
miR5244	UGUCUGAUGAAGAUUGUUGGU	21	No		Alfalfa
miR5499	AAGGAAGAAUCAGUUAUGUACA	22	No		Rice
miR6300	GUCGUUGUAGUAUAGUGGUGA	21	No		Soybean
miR6475	UCUUGAGAAGUAGAGAACCGACAG	24	No		Populus trichocarpa
miR6478	CCGACCUUAGCUCAGUUGGUA	21	No		Populus trichocarpa
miR7502	UAACGGUAGAAGAAGGACUGAA	22	No		Cotton
miR7696	UUGAAUUAUGCAGAACUUAUCA	22	No		Alfalfa
miR8175	CGUUCCCCGGCAACGGCGCCA	21	No		Arabidopsis
miR9666	CGGUAGGGCUGUAUGAUGGCGA	22	No		Wheat
Peanut-specific	Mature sequence	Length(nt)	Star(*)	References	Conservative in other plants
miR3508	UAGAGGGUCCCCAUGUUCUCA	21	No	Zhao et al., 2010 [24]; Chi et al., 2011 [35].	Peanut
miR3509	UGAUAACUGAGAGCCGUUAGAUG	23	Yes	Zhao et al., 2010 [24].	Peanut
miR3511	GCCAGGGCCAUGAAUGCAGAA	21	No	Zhao et al., 2010 [24].	Peanut
miR3512	CGCAAAUGAUGACAAAUAGACA	22	No	Zhao et al., 2010 [24].	Peanut
miR3513	UGAUAAGAUAGAAAUUGUAUA	21	Yes	Zhao et al., 2010 [24].	Peanut
miR3514	UCACCGUUAAUACAGAAUCCUU	22	Yes	Zhao et al., 2010 [24].	Peanut
miR3515	AAUGUAGAAAAUGAACGGUAU	21	No	Zhao et al., 2010 [24].	Peanut
miR3516	UGCUGGGUGAUAUUGACAGAA	21	No	Zhao et al., 2010 [24].	Peanut
miR3517	UCUGACCACUGUGAUCCCGGAA	22	No	Zhao et al., 2010 [24].	Peanut
miR3518	GACCUUUGGGGAUAUUCGUGG	21	No	Zhao et al., 2010 [24].	Peanut
miR3519	UCAAUCAAUGACAGCAUUUCA	21	No	Zhao et al., 2010 [24].	Peanut
miR3520	AGGUGAUGGUGAAUAUCUUAUCUU	24	No	Zhao et al., 2010 [24].	Peanut
Novel	Mature sequence	Length(nt)	Star(*)	References	Conservative in other plants
miRn1	UUCCCAAUUCCACCCAUUCCUA	22	Yes		
	UUUUCCCAAUUCCACCCAUUCC	22	Yes		
miRn2	UUUUCAUUCCAUACAUCAUCUA	22	Yes		
	UUUUCAUUCCAUACAUCAUCU	21	Yes		
	UUUCAUUCCAUACAUCAUCUA	21	Yes		
miRn3	UAGAGGGUCCCCAUGUUCUCA	21	Yes		
miRn4	UGAAGCAAAGUGAUGACUCUG	21	Yes		
miRn5	UGUGUGGGUUUCUGGUCUCCAC	22	Yes		
miRn6	AUCCCUCGAAGGCUUCCGCUA	21	Yes		
miRn7	UUAUUGUCGGACUAAGGUGUCU	22	Yes		
miRn8	UUGAUGCAGUACGGACAAAAG	21	Yes		
miRn9	UUUGUGUGAAAGAUCUCCGGA	21	No		
miRn10	CGGUUGUGUGGAGUGCUACGG	21	No		
miRn11	AGGUGCCGGUGCAUUUGCAGG	21	No		
miRn12	AUGAGCUCAGUUGAAGAUUUG	21	Yes		
miRn13	GGAACAAAGAGUUUGAGAUGG	21	Yes		
miRn14	AAAUUGAUUGAUUAUUCCUGA	21	Yes		
miRn15	UUGCUAGGAUCGUUUGGCGAU	21	Yes		
miRn16	UGCUUAGGAAGGAUUGUCUUA	21	Yes		
miRn17	AGGGCGUUAUGUAGGGCAUC	20	No		
miRn18	UUGGUAGUAGAAGAAGGAGAU	21	No		
miRn19	UCUGAAUGGGAUGAAAACGCU	21	No		
miRn20a	UUUGGAAAUUCGGUACAUUAA	21	Yes		
miRn20b	UUUGGAAACUCGGUACAUUAA	21	Yes		
miRn21	UUACGUGUACACAAAAAAUCA	21	No		
miRn22	UGAAAGUGGAAUUAAAGCAAG	21	No		
miRn23	GUCGACUUUACAUGAAGUUGA	21	Yes		
miRn24	UUUGGGUCUUGAGAGUACAUG	21	No		

* means star sequence of miRNA

### Differential expression of miRNAs during peanut pod development

**Fig. 2:**
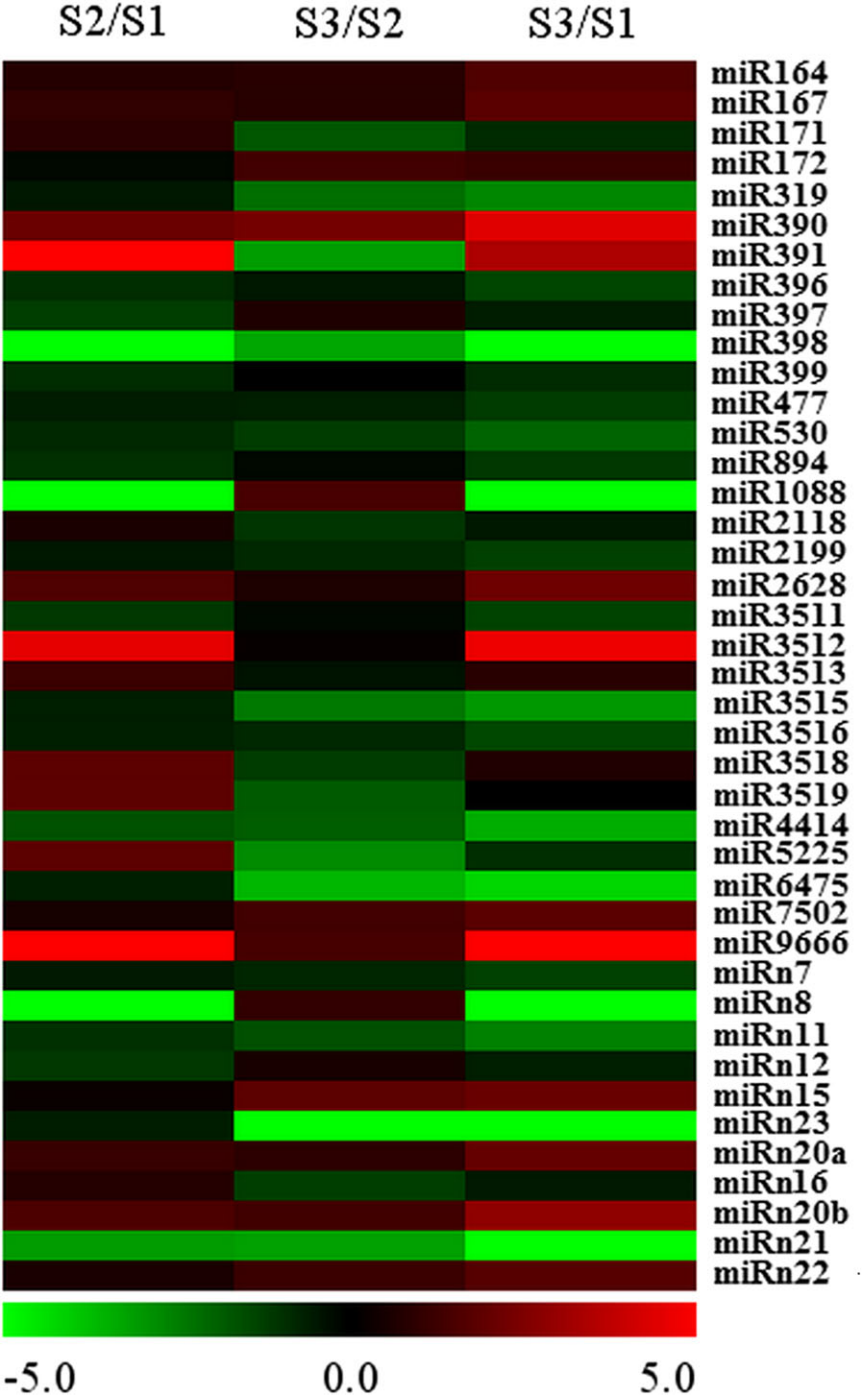
Clustering and differential expression analysis of miRNAs across S1, S2 and S3 using deep sequencing data. Data was presented as log_2_fold change by comparing miRNA abundances (TPM) between S2 and S1, S3 and S2, S3 and S1

**Fig. 3:**
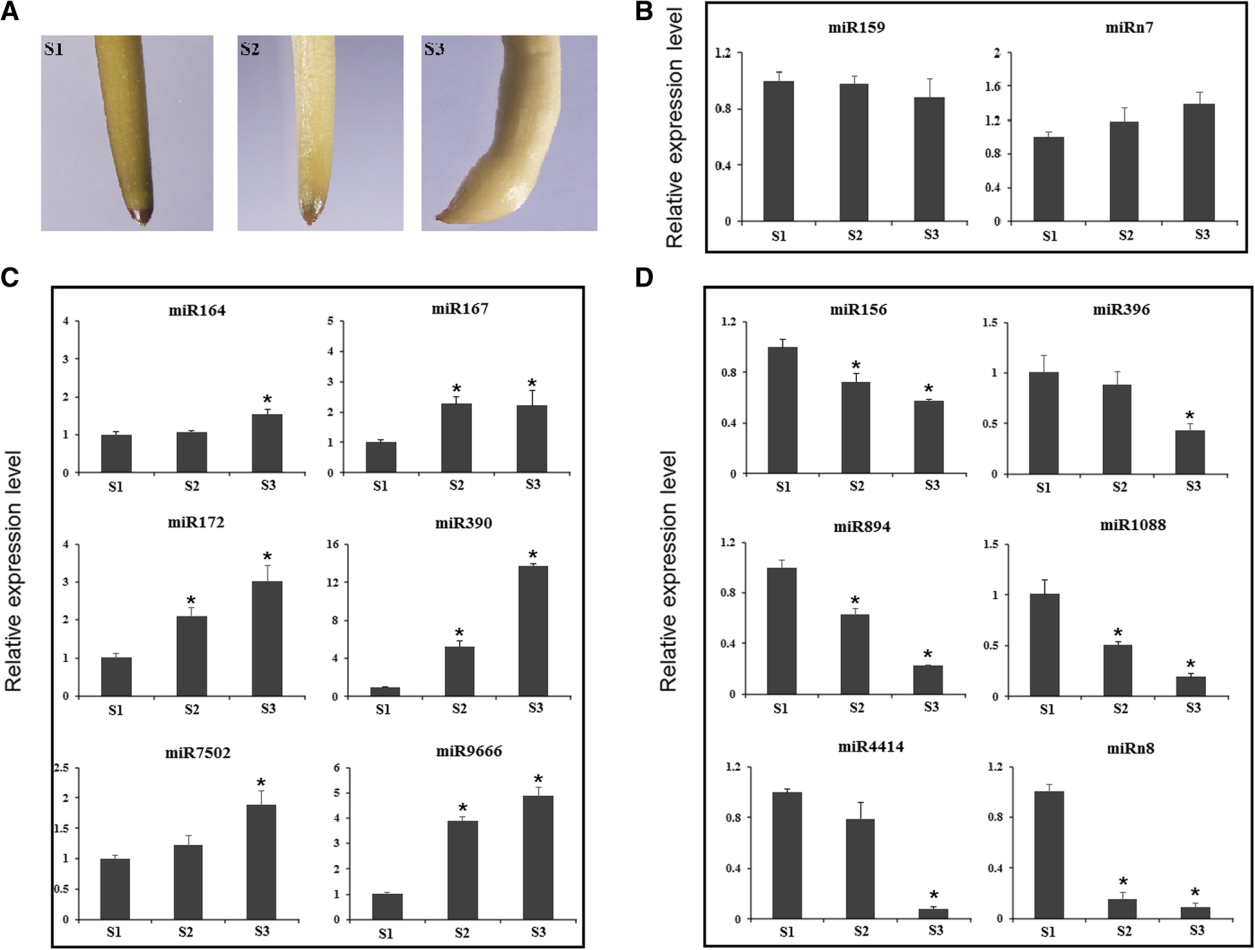
Expression analysis of pod development-related miRNAs using qRT-PCR. **a** Phenotype of gynophore in S1, S2 and S3. **b** MiRNAs with similar expression level at S1, S2 and S3 gynophores. **c** Up-regulated miRNAs during pod development. **d** Down-regulated miRNA during pod development. Error bars indicate SD of three biological replicates. *Asterisk* indicated a statistically significant difference (*P* < 0.05)

After normalization, we analyzed the expression pattern of all miRNAs identified in this study (for detailed statistics analysis of all miRNAs, see Additional file 5: Table S2). In total, 40 miRNA families exhibited differential accumulation during early pod development. Of them, 15 known miRNA families and four novel miRNA families were differentially expressed between S1 and S2, whereas 16 known miRNA families and seven novel miRNA families showed different expression between S2 and S3 (Fig. 2). 22 known miRNA families and nine novel miRNA families showed differential accumulation between stages S1 and S3 (Fig. 2). To validate the sequencing data, qRT-PCR was performed to examine the expression of several miRNAs that maybe related to early pod development and the results were in agreement with the sequencing data except for miRn7 where the sequencing result and qRT-PCR showed different patterns (Fig. 3b). As shown in Fig. 3c, miR164, miR167, miR172, miR390, miR7502 and miR9666 were up-regulated significantly, while miR156, miR396, miR894, miR1088, miR4414 and miRn8 were significantly down-regulated during early pod development (Fig. 3d). Different accumulation levels of miRNAs between different developmental stages suggested a possible miRNA-mediated regulation of gene expression during peanut embryo and early pod development in a temporal manner.

### Degradome sequence analysis and target gene identification

To gain a better understanding of the regulatory role of miRNAs during peanut early pod development, it is necessary to identify their target genes that could provide valuable information for miRNA function during this process. Two degradome libraries from gynophores that unburied and buried in soil for about three days (named as D1 and D2) were constructed separately. By sequencing these two libraries, 17.2 and 23.8 million clean reads were obtained and more than 99% of the sequences were 20 or 21 nt in length. In total, 3,896,267 (53.34%) and 4,600,466 (52.95%) unique reads were mapped to peanut cDNAs which were subjected to target identification (Additional file 6: Table S5). The cleaved transcripts were categorized into three classes (Class 0, 1 and 2), as reported previously [29]. Class 0 transcripts contained only one maximum peak from miRNA-directed cleavage, representing perfect data with no other contamination. Class 1 transcripts contained more than one maximum peaks and the miRNA cleaved peaks are equal to the maximum. Class 2 was transcripts with the peaks from miRNA-directed cleavage lower than the maximum. In this study, a total of 105 target genes for 40 known miRNA families and 10 target genes for seven novel miRNA families were identified (Table 2). Among the 115 identified targets, 79 targets (71%) belonging to class 0, whereas 17 and 19 were classified into class 1 and Class 2, respectively. Most of the cleavage sites were located in CDS region, and only a few cleavage sites were located in 5′-UTR or 3′-UTR (Table 2). The abundance of cleaved transcripts was normalized using ‘reads per 10 million’ (RP10M) method. Interestingly, the cleaved transcripts of many target genes were differently accumulated between these two libraries (Table 2), providing important evidence for miRNA function during early pod development in peanut.

**Table 2:** miRNA-mRNA target pairs identified in at least one library of peanut gynorphore with p-value ≤ 0.05

miRNA	Target gene	Target annotation	Cleavage site(nt)	Target site location	Class	Abundance in D1 (RP10M)	Abundance in D2 (RP10M)
ahy-miR156/157	Araip.QT8AK.1	Squamosa promoter binding protein	999	CDS	0	93	54
	Araip.0ZF73.1	Squamosa promoter binding protein	2069	3′-UTR	0	27	28
	Araip.II3JP.1	Squamosa promoter binding protein	1557	3′-UTR	0	628	127
	Araip.RD4BX.1	Squamosa promoter binding protein	1423	3′-UTR	0	64	124
ahy-miR159	Araip.B1U9G.1	MYB transcription factor	661	CDS	0	7	33
	Araip.0279H.1	MYB transcription factor	1506	CDS	0	2	3
ahy-miR160	Araip.NYM6Q.1	Auxin response factor	1364	CDS	0	67	46
	Araip.BYV33.1	Auxin response factor	1429	CDS	0	257	430
	Araip.18474.1	Digalactosyldiacylglycerol synthase	1629	CDS	1	2	0
	Araip.9V70N.1	Solute carrier	955	CDS	0	15	72
ahy-miR164	Araip.D25HB.1	Transcriptional factor NAC	786	CDS	0	60	130
	Araip.DLE10.1	Heat shock protein	580	CDS	1	10	12
ahy-miR166	Araip.IIT7G.1	ARF GTPase-activating protein	1048	CDS	0	4	3
	Araip.P57FD.1	Serine/threonine kinase	552	CDS	0	10	40
	Araip.80WHH.1	Peroxidase	828	CDS	1	11	5
	Araip.8GH41.1	Plastidic glucose transporter	120	CDS	0	2	10
ahy-miR167	Araip.7M6VI.1	Pentatricopeptide repeat protein (PPRP)	1005	CDS	0	0	118
	Araip.R1QSY.1	Auxin response factor	568	CDS	2	0	2
ahy-miR168	Araip.FPV8R.1	Argonaute protein 1	379	CDS	0	113	188
ahy-miR169	Araip.T3WCA.1	Nuclear transcription factor Y	971	3′-UTR	0	45	130
ahy-miR171	Araip.E00UL.1	Transcription factor GRAS	542	CDS	0	209	845
	Araip.27I5U.1	Gibberellin receptor	290	CDS	1	0	5
ahy-miR172	Araip.Y07A4.1	Ethylene-responsive transcription factor AP2	1253	5′-UTR	0	476	965
	Araip.AE7EH.1	Cell division protease	825	CDS	0	4	1
	Araip.HRN64.1	Embryogenesis abundant protein	55	5′-UTR	1	3	7
ahy-miR319	Araip.SZ7Q5.1	Monodehydroascorbate reductase	1263	CDS	0	88	52
	Araip.Z17TF.1	Transcription factor TCP	1688	CDS	0	41	55
	Araip.KK7TK.1	DELLA protein	939	CDS	1	0	3
ahy-miR390	Araip.VT2PQ.1	TAS3	342		0	0	25
	Araip.43TDN.1	Solute carrier family 50 (sugar transporter)	96	CDS	0	1	45
ahy-miR391	Araip.5A463.1	Aluminium induced protein	309	CDS	2	6	15
	Araip.HW8E3.1	Homeobox-leucine zipper protein	2183	CDS	2	2	2
	Araip.65E1M.1	LA RNA-binding protein	2280	CDS	2	0	2
ahy-miR393	Araip.774UX.1	Auxin signaling F-box protein	1906	CDS	0	589	968
	Araip.0E25I.1	Auxin signaling F-box protein	1710	CDS	0	296	429
	Araip.0XA60.1	Auxin signaling F-box protein	297	CDS	2	4	5
	Araip.NRD2A.1	Brassinosteroid receptor kinase	79	CDS	2	0	3
ahy-miR394	Araip.Y8EUA.1	Glutathione S-transferase	760	CDS	0	5	0
ahy-miR395	Araip.BC9AA.1	Cellulose synthase	1531	CDS	0	6	3
ahy-miR396	Araip.6YN77.1	Growth-regulating factor	922	CDS	0	422	97
	Araip.SE9FW.1	MADS-box transcription factor	313	CDS	0	0	11
ahy-miR397	Araip.QA79V.1	Laccase 10 (lignin catabolic process)	743	CDS	0	8	5
	Araip.Z5USZ.1	Laccase 11 (lignin catabolic process)	746	CDS	0	3	2
ahy-miR398	Araip.SEZ68.1	Calcium-dependent protein kinase	1309	CDS	1	2	0
ahy-miR399	Araip.G5CUD.1	Disease resistance protein	4095	CDS	0	2	24
	Araip.RXA31.1	Expansin-A4(cell wall organization)	958	5′-UTR	0	8	4
	Araip.I7ZGU.1	Unknown protein	173	CDS	0	8	11
ahy-miR414	Araip.IJ273.1	Ribosome biogenesis protein	1818	CDS	0	8	20
	Araip.467MQ.1	Serine/threonine-protein phosphatase	1586	CDS	0	10	13
	Araip.HJ37G.1	Phosphoinositide phospholipase C	1118	CDS	0	19	24
	Araip.Y25R8.1	ARF guanine-nucleotide exchange factor	755	CDS	0	23	38
	Araip.98Q8H.1	Sequence-specific DNA binding transcription factor	359	CDS	0	7	0
	Araip.FPJ1M.1	DDB1-CUL4 associated factor (protein binding)	5387	CDS	0	0	11
ahy-miR477	Araip.6IZ1V.1	Mitogen-activated protein kinase	147	CDS	0	2	2
	Araip.48K15.1	Heat shock cognate protein	190	CDS	2	7	7
	Araip.A6M6K.1	Cytosolic ascorbate peroxidase	163	CDS	2	30	37
	Araip.BP9MY.1	Myo-inositol-1-phosphate synthase	34	5′-UTR	0	0	15
	Araip.N0DQ0.1	Dual specificity protein phosphatase	33	5′-UTR	0	0	2
	Araip.N1PSJ.1	Glutamate synthase	22	CDS	0	0	5
	Araip.SJE6C.1	Unknown protein	64	CDS	0	0	16
ahy-miR482	Araip.61H7R.1	WD repeat-containing protein	57	CDS	0	4	16
	Araip.9T0HK.1	E3 ubiquitin-protein ligase	1029	CDS	0	48	44
	Araip.313YK.1	E3 ubiquitin-protein ligase	4548	CDS	2	0	3
	Araip.6W5RU.1	E3 ubiquitin protein ligase	1890	CDS	2	0	3
	Araip.NX28V.1	Disease resistance protein	3219	3′-UTR	2	0	4
	Araip.BX1V3.1	Disease resistance protein	4147	CDS	2	0	2
ahy-miR530	Araip.87MXF.1	Nuclear protein required for cytoskeleton organization	2092	CDS	1	7	0
	Araip.X3V04.1	Unknown protein	481	CDS	0	6	0
	Araip.YGJ1S.1	Leucine-rich repeat receptor kinase	1659	CDS	0	0	2
ahy-miR1088	Araip.6BJ8Z.1	Pentatricopeptide repeat protein (PPRP)	27	5′-UTR	0	113	0
ahy-miR1507	Araip.UGA40.1	LRR-NB-ARC domain disease resistance protein	4474	CDS	0	148	119
	Araip.SJE6C.1	DUF4228 domain protein	64	5′-UTR	0	11	16
	Araip.4Q4DB.1	NBS-LRR domain disease resistance protein	1036	CDS	0	21	0
	Araip.AX6A6.1	Disease resistance protein	880	CDS	0	14	0
	Araip.KW5UK.1	Disease resistance protein	939	CDS	0	14	0
	Araip.L51CJ.1	Disease resistance protein	880	CDS	0	14	0
ahy-miR1511	Araip.08W0L.1	Protein binding protein	174	CDS	0	0	2
	Araip.PD52B.1	Aluminum sensitive protein	345	CDS	0	9	10
ahy-miR1515	Araip.9C688.1	Chlorophyll a-b binding protein	375	CDS	1	17	1
	Araip.U8QGY.1	DNA-lyase-like isoform	963	CDS	0	0	9
	Araip.5RQ6Z.1	ATP binding protein	3153	CDS	0	32	0
ahy-miR1520	Araip.AW9H3.1	DNA methyltransferase	687	CDS	0	2	0
	Araip.5K8JY.1	DNA methyltransferase	576	CDS	1	3	0
	Araip.LK5X5.1	Protein kinase	2471	CDS	2	0	5
	Araip.U0MS2.1	Zinc finger CCCH domain-containing protein	1559	3′-UTR	2	0	2
ahy-miR2111	Araip.KV8TN.1	Ubiquitin carboxyl-terminal hydrolase	79	CDS	0	5	1
	Araip.8SC4I.1	Anaphase-promoting complex subunit	426	CDS	1	2	0
	Araip.QW087.1	Dihydroorotate dehydrogenase	46	5′-UTR	0	0	2
ahy-miR2118	Araip.B2Q36.1	Translation initiation factor eIF	1643	CDS	0	38	18
	Araip.QG6DX.1	Zinc finger protein	3535	CDS	0	4	0
	Araip.NP9KT.1	Carboxylate dehydrogenase	1932	3′-UTR	2	2	4
	Araip.E41BL.1	Disease resistance protein	344	CDS	0	0	9
	Araip.IKJ6N.1	Disease resistance protein	666	CDS	2	0	4
ahy-miR2199	Araip.I1L37.1	bHLH transcription factor	741	CDS	0	593	312
ahy-miR2628	Araip.LY8H2.1	Protein kinase	55	5′-UTR	0	24	22
ahy-miR3514	Araip.GQ8VC.1	Pentatricopeptide repeat protein (PPRP)	360	CDS	0	2699	435
	Araip.N7IGZ.1	Pentatricopeptide repeat protein (PPRP)	1134	CDS	0	53	4
ahy-miR4376	Araip.0K3S5.1	Cullin-like protein	2083	CDS	2	2	0
	Araip.Q4HT6.1	Methyltransferase	2372	CDS	0	9	0
	Araip.NS167.1	ATP-dependent RNA helicase-like protein	2264	CDS	0	0	8
ahy-miR4414	Araip.B5L53.1	DNA binding transcription factor	989	CDS	1	2	0
ahy-miR5021	Araip.166TL.1	MADS-box transcription factor	189	5′-UTR	0	3	0
ahy-miR5225	Araip.WG5D6.1	Histidine kinase	180	CDS	1	0	2
ahy-miR6300	Araip.VJ5LB.1	Dehydroascorbate reductase	70	CDS	2	62	198
ahy-miR9666	Araip.99HRK.1	Protein kinase (protein ubiquitination process)	537	CDS	1	2	0
ahy-miRn1	Araip.9T0HK.1	E3 ubiquitin protein ligase	1260	CDS	0	45	43
	Araip.71CS3.1	Transcriptional factor NAC	1420	CDS	0	81	241
	Araip.QG6DX.1	Zinc finger protein	3535	3′-UTR	0	4	0
ahy-miRn2	Araip.C0ZFN.1	LRR-NB-ARC domain disease resistance protein	704	CDS	0	17	45
	Araip.I8IY2.1	TIR-NBS-LRR domain disease resistance protein	3782	CDS	1	17	0
ahy-miRn4	Araip.JPM97.1	Receptor kinase	533	CDS	0	4	9
ahy-miRn5	Araip.3TX6Y.1	Unknown protein	300	CDS	0	0	5
ahy-miRn7	Araip.K65JZ.1	Major intrinsic protein (transporter activity)	189	CDS	0	319	1084
ahy-miRn10	Araip.T99DR.1	DNA polymerases (DNA replication)	20	CDS	1	11	0
ahy-miRn24	Araip.NF709.1	Cytochrome P450 protein	386	CDS	0	4	9

*RP10M* reads per 10 million clean reads

**Fig. 4:**
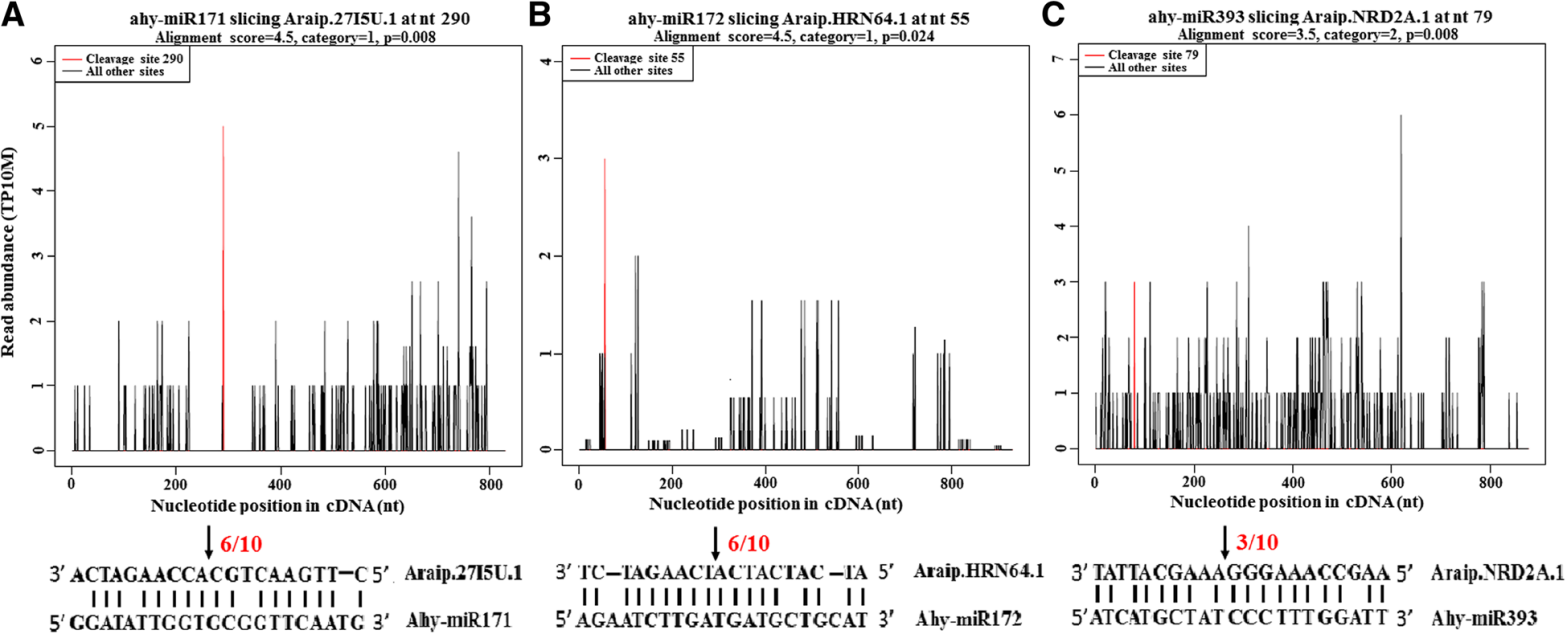
Target plots of newly identified target genes for known miRNAs and target validation by RLM 5′-RACE. Peaks in red are the signatures produced by miRNA-directed cleavage. **a** Cleavage features in gibberellin receptor mRNA by miR171. **b** Cleavage features in embryogenesis abundant protein mRNA by miR172. **c** Cleavage features in brassinosteroid receptor kinase mRNA by miR393. RLM 5′-RACE was used to verify the cleavage sites in gibberellin receptor gene, embryogenesis abundant protein gene and brassinosteroid receptor kinase gene by miR171, miR172 and miR393. The numbers above the *vertical arrow* indicate the number of sequences found at the exact cleavage site

Most targets of the conserved miRNAs were transcription factors such as the squamosa promoter binding-Like (SPL), TEOSINTE BRANCHED1/CYCLOIDEA/PRO-LIFERATING CELL FACTOR1 (TCP), MYB, ARF, NAC, GRAS and AP2, which have been identified previously in diverse plant species [30, 31]. MiR399, miR482, miR1507 and miR2118 have been found to target disease resistance protein genes, and miR168 was found to target gene encoding AGO1, a key component of RNA-induced silencing complex (RISC). Additionally, one *TAS3* was identified as target of miR390 that give rise to the production of phased ta-siRNAs from that precursor. For the less conserved miRNAs, the targets were not enriched in transcription factors and were more likely to be involved in metabolic and signal transduction. Most peanut-specific miRNAs did not have detectable sliced targets in the degradome libraries. Only two genes encoding pentatricopeptide repeat protein (PPRP) were detected as miR3514 targets. This may be explained by the low abundance of these miRNAs or their sliced targets in peanut gynophore. In addition to the previously identified conserved targets we also identified many new targets in peanut for the known miRNAs. These putative novel targets include digalactosyldiacylglycerol synthase and solute carrier (miR160), heat shock protein (miR164), serine/threonine kinase (miR166), PPRP (miR167), DNA methyltransferase (miR1520 and miR4376) and others (Table 2). Interestingly, one embryogenesis abundant protein gene was shown to be target of miR172, one GA receptor gene and one BR receptor kinase gene were shown to be target of miR171 and miR393. These results were independently verified by RLM 5′-RACE analysis (Fig. 4). These conserved miRNAs regulated non-conserved targets in addition to the conserved targets may be specific to peanut and play important roles in pod development. As shown previously in soybean and tomato, the targets of novel miRNAs were not enriched in transcription factors [26, 31]. The present data confirmed these results. Among the 10 targets of novel miRNAs, only one target encoding transcriptional factor (miRn1). Two targets of miRn2 are involved in disease resistance (Table 2). Meanwhile, novel miRNAs targeted a number of functional genes, such as E3 ubiquitin-protein ligase gene (miRn1), major intrinsic protein gene (miRn7), DNA-directed DNA polymerase gene (miRn10) and cytochrome P450 gene (miRn24). However, the function of these newly identified targets, and their regulation by miRNA in peanut pod remains to be determined.

### GO enrichment and KEGG pathway analyses of target genes

**Fig. 5:**
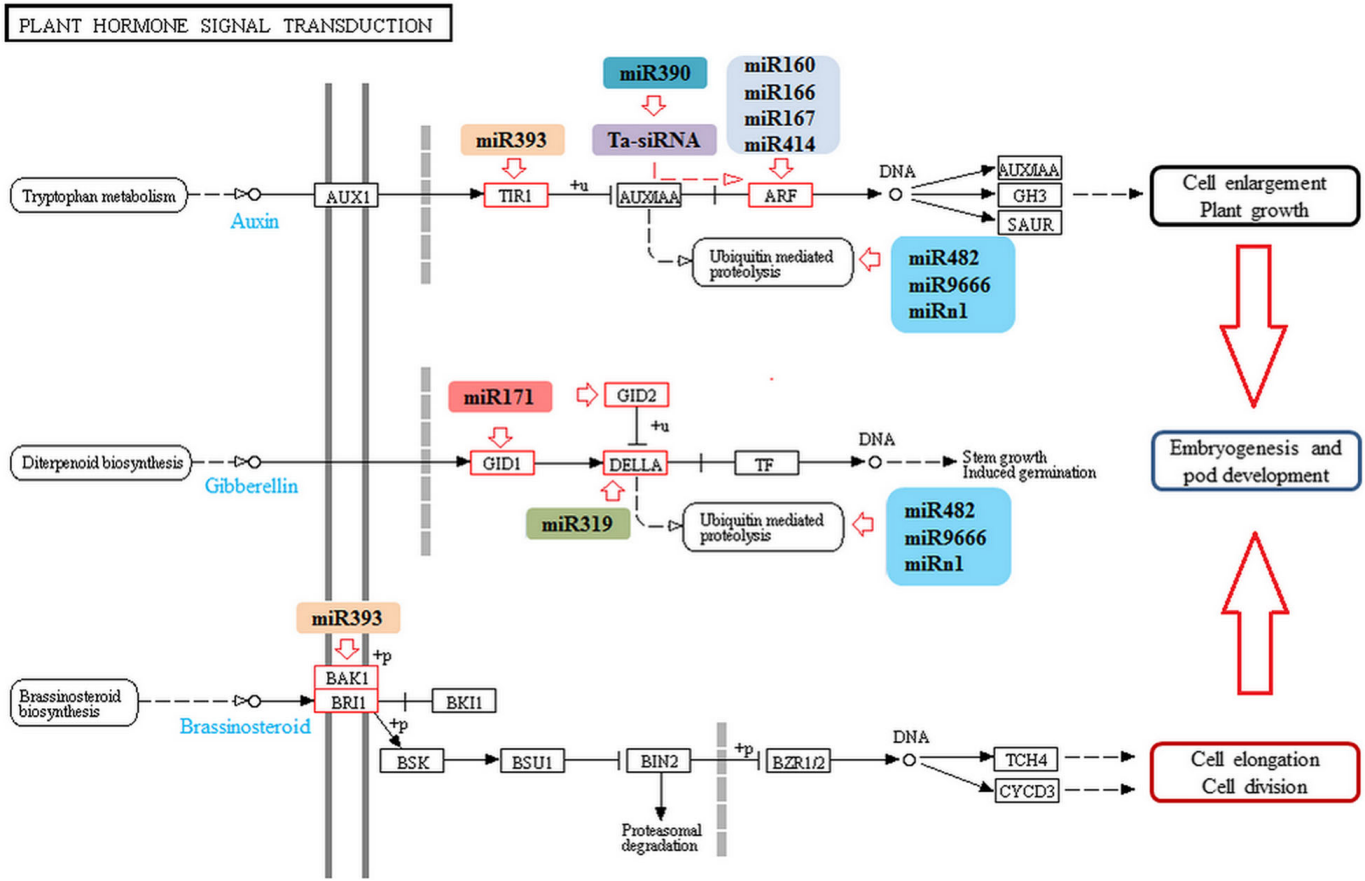
KEGG pathways related to plant hormone signal transduction targeted by peanut miRNAs

All 115 target genes identified in this study were subjected to Gene Ontology (GO) functional classification and Kyoto Encyclopedia of Genes and Genomes (KEGG) analysis to perceive their biological roles using WEGO toolkit [32]. A total number of 89 miRNA targets could be annotated by GO classification. It was determined that these target genes were involved in seven types of cellular component, six types of molecular function and 14 types of biological process with the cell part, binding and metabolic process were the most abundant groups in each category (Additional file 7: Figure S3A). According to KEGG analysis, 52 target genes were significantly enriched in 11 pathways including plant hormone signal transduction, ascorbate and aldarate metabolism, plant-pathogen interaction, metabolic pathways and ribosome biogenesis (Additional file 7: Figure S3B). Plant hormone signal transduction pathway and the corresponding miRNAs are shown in Fig. 5. In this pathway, 12 genes are targeted by seven miRNAs. In addition, miR390 targets ARF genes indirectly by giving rise to the formation of ta-siRNAs [33]. Moreover, three miRNAs (miR482, miR9666 and miRn1) are involved in ubiquitin-mediated proteolysis process by targeting E3 ubiquitin ligase gene, through which control the protein accumulation levels of AUX and DELLA in IAA and GA pathways, respectively. These findings highlight the significant regulation of miRNAs on peanut early pod development by effecting hormone signaling transduction pathways.

### Correlated analysis between miRNAs and target mRNAs during early pod development

**Fig. 6:**
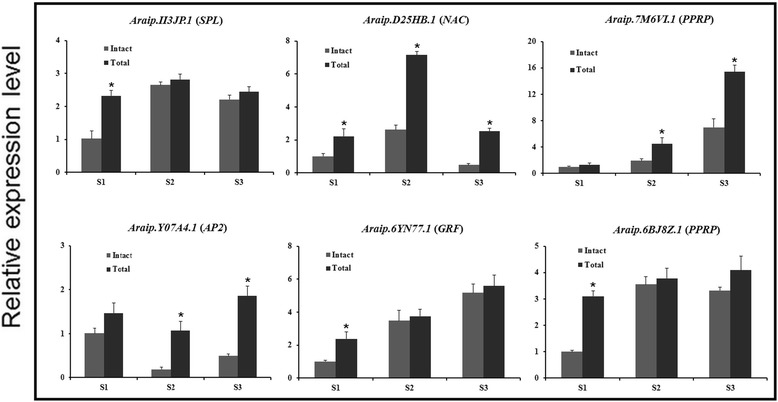
qRT-PCR analysis of total and intact mRNA levels in peanut gynophore using two different primers sets. *Error bars* indicate the SD of three biological replicates. The *asterisk* indicated a statistically significant difference (*P* < 0.05)

Integrated analysis of miRNAs and their targets expression can help to understand the regulatory pathways of miRNAs and identify functional miRNA-mRNA modules involved in peanut embryo and early pod development. Here, we profiled the accumulation of six target mRNAs validated by degradome sequencing in peanut gynophore using qRT-PCR. To determine exactly how much of the mRNA were cleaved by miRNA, we detected the total mRNA and the intact mRNA that uncleaved by miRNA using two pairs of primers designed in the 3′-UTR region and spanning the miRNA target site, respectively [31]. As shown in Fig. 6, the total mRNA of all the target genes increased during early pod development and the intact mRNA were also increased except for *AP2* which is targeted by miR172. Meanwhile, increased cleavage of the *NAC*, *PPRP* and *AP2* transcripts (targeted by miR164, miR167 and miR172, respectively) and decreased cleavage of the *GRF* and another *PPRP* transcripts (targeted by miR396 and miR1088, respectively) were observed at S2 and S3 compared with S1 stage, which in agreement with the miRNA expression profiling that miR164, miR167 and miR172 were up-regulated while miR396 and miR1088 were decreased during peanut early pod development. These results suggested that miRNA significantly modulate their intact target mRNAs accumulation at the post-transcriptional level to regulate them at appropriate expression levels, controlling peanut early pod development.

## Discussion

### Roles of miRNAs during peanut embryogenesis and early pod development

The early stage of peanut pod development including gynophore elongation, pod enlargement, cell differentiation and embryogenesis is a complicated biological process regulated by coordinated gene expression. Increasing evidence indicated that miRNAs play important regulatory roles in cell differentiation and plant development. However, the function of miRNAs during peanut embryogenesis and early pod development has not been addressed. In previous reports, Zhao and Chi identified 22 and 33 known miRNA families from libraries constructed using mixed RNAs from peanut root, stem, leave and seed, respectively [23, 34]. In the current study, deep sequencing of small RNA libraries constructed using peanut S1, S2 and S3 gynophore RNAs led to the discovery of 69 known miRNA families and 24 novel miRNA families. Interestingly, 34 known miRNA families were first identified in peanut, suggesting that they were preferentially expressed and specific to peanut gynophore or young pod. Among them, 10 known but less conserved miRNAs(miR1520, miR2199, miR2628, miR4414, miR5221, miR5227, miR5234, miR5244, miR6300 and miR7696) were only identified in leguminous plants [35, 36]. In addition, 12 known but non-conserved miRNAs were also detected in peanut gynophore with a lower abundance than that of conserved miRNAs. It has been proposed that conserved miRNAs are probably responsible for regulation of the basic cellular and developmental processes, while the species-specific miRNAs are involved in the regulation of species-specific regulatory pathways [37, 38]. These legume- or peanut-specific miRNAs may function in regulation of gene expression during peanut- or pod-specific processes. Interestingly, miRn8 accumulated only in S1 stage and peanut-specific miR3512 expressed only in S2 and S3 stages, indicating that they act in a tissue- or cell- specific manner and may play essential roles in peanut embryo and early pod formation.

### Putative mRNA-miRNA modules involved in peanut early pod development

To further explore the regulatory roles of miRNAs during peanut embryogenesis and early pod development, we profiled their differential expression among three developmental stages. Based on the normalized abundance of high-throughput sequencing data, 40 miRNA families were differentially accumulated during early pod development which may contribute to cell proliferation and differentiation during embryogenesis and early developmental stage of peanut pod. A large number of conserved target genes for differentially expressed miRNAs were identified, such as SPL, MYB, ARF, NAC, NF-Y, GRAS, AP2 and TCP type transcription factors, which have been experimentally validated by previous studies [30, 31]. Based on the normalized abundance of degradome sequencing data, miR156-mediated cleavage of *SPL* (*Araip.II3JP.1*), miR164-mediated cleavage of *NAC* (*Araip.D25HB.1*), miR167-mediated cleavage of *PPRP* (*Araip.7M6VI.1*), miR171-mediated cleavage of *GRSA* (*Araip.E00UL.1*), miR172-mediated cleavage of *AP2* (*Araip.Y07A4.1*), miR393-mediated cleavage of F-box gene (*Araip.774UX.1*), miR396-mediated cleavage of *GRF* (*Araip.6YN77.1*) and miR1088-mediated cleavage of another *PPRP* (*Araip.6BJ8Z.1*) were the most abundant and differently accumulated between the two degradome libraries. These miRNA-mRNA modules might be involved in regulating biological processes that facilitate peanut embryogenesis and pod development. Indeed, miR156-mediated regulation of *SPL* transcripts has been proved to play critical roles in regulating zygotic embryo development in *Arabidopsis* [39]. MiR164-mediated suppression of *NAC* is required for embryogenesis, shoot meristem development, lateral root formation, senescence and other developmental processes [40]. Our results showed that miR156-directed cleavage of *SPL* declined whereas miR164-directed cleavage of *NAC* transcripts increased during early pod development (Fig. 6), which consists with the earlier observed expression profiles of miR156 and miR164 determined by qRT-PCR (Fig. 3). Moreover, a large number of new targets were also detected for conserved as well as non-conserved miRNAs, although splicing frequency of these new targets was very low. For example, one embryogenesis abundant protein gene emerged as the target of miR172. Three miRNAs (miR167, miR1088 and miR3514) target genes encoding PPRP. PPRP has been demonstrated to play important roles in the first mitotic division during gametogenesis and in cell proliferation during embryogenesis [41]. These results suggested the present of non-conserved miRNA-mRNA modules that were specific to peanut and play crucial roles in regulating peanut-specific biological processes that promote embryo and early pod development.

### Network consist of hormone, light signal and miRNAs in regulating peanut embryo and early pod development

**Fig. 7:**
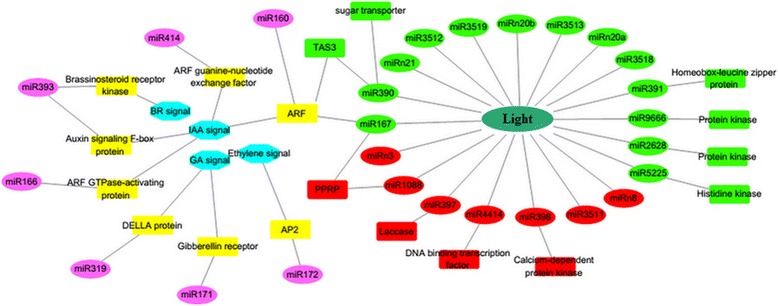
Regulatory network consist of light, miRNAs and hormone during peanut embryo and early pod development. In green ovals are up-regulated miRNAs and in red ovals are down-regulated miRNAs under dark condition

Peanut is a typical ‘aerial flower and subterranean fruit’ plant, and peanut fruit completes the development process under ground. After fertilization, peanut zygote divides few times and then the embryonic development stops when exposed to light condition or normal day/night period. Along with the elongation of gynophore, the tip region (containing the embryo) of gynophore is buried into soil, peanut embryogenesis and pod development resumes in the darkness, indicating that light is an important environmental signal that regulates pod formation and development. Physiological studies demonstrated that red light and white light inhibited the growth of peanut ovules [6, 42]. Besides, multiple hormonal pathways are often modulated by light signal to control diverse developmental processes. Given that the critical roles of miRNA on plant embryogenesis, dissect the crosstalk among light signal, endogenous hormones and miRNAs would be of great interest. Our results showed that the expression of many known and novel miRNAs that involved in embryo development was affected by light signal through profiling analysis between S1 (light condition) and S2 (dark condition) such as miR167, miR390 and miR1088 (Fig. 7). MiR167 and miR1088 mediated *PPRP* cleavage as well as miR390 mediated *ARF* cleavage were known to participate in embryogenesis [41, 43]. This result suggests that miRNA might be a molecular integrator that link light signaling to the multiple hormone pathways such as auxin.

Plant endogenous hormones play vital roles in diverse developmental processes. For instance, GA can regulate gene expression to control stem elongation, seed germination and embryo development in plants [44–46]. Auxin is considered to be the main hormone involved in plant differentiation through controlling cell polarity, cell division and cell elongation [43, 47]. Furthermore, miRNA regulation of auxin pathway plays an important role during cotton somatic embryogenesis [48]. In peanut gynophore, either the content or the distribution patterns of IAA, GA and BR significantly changed from S1 to S3, suggesting that these hormones are key regulators of peanut embryo development and pod formation [12, 49]. Here, it was found that eight target genes that participate in auxin signal transduction, two genes that participate in GA signal transduction and one gene that participates in BR signal transduction were identified as miRNA targets through degradome sequencing analysis (Fig. 5). In addition, we also found that miR390 could mediate the cleavage of *TAS3* in peanut. The cleavage of *TAS3* by miR390 could induce the formation of phased ta-siRNAs that mediate the regulation of auxin signal and in turn influence diverse developmental processes in flowering plants [33, 50]. Profiling analysis showed that several miRNAs (miR167, miR319 and miR390) participating in auxin and GA signal transduction pathways were differentially accumulated during peanut pod development. These differentially expressed miRNAs and their hormone-related targets might be essential components of the regulatory networks in peanut embryogenesis and early pod development (Fig. 7). Collectively, miRNAs, hormones and light signal comprises a complex network regulating specific biological processes controlling peanut embryo and pod development.

## Conclusions

High-throughput sequencing together with bioinformatics and experimental approaches were used to explore the function of miRNAs in peanut embryogenesis and early pod development. A total of 70 known and 24 novel miRNA families were discovered. Among them, many miRNAs were legume-specific or peanut-specific and differentially expressed during early pod development. In addition, 115 target genes were identified for 47 miRNA families. Several new targets that might be specific to peanut were found and further validated by RLM 5′-RACE. These peanut-specific and differentially expressed miRNAs and their corresponding target genes might be essential components of the regulatory networks controlling in peanut embryogenesis and early pod development.

## Methods

### Plant materials and growth conditions

Plant materials were collected from cultivated peanut (Luhua-14) grown in the experimental farm of Shandong Academy of Agricultural Sciences with normal day/night period. The gynophores were staged based on developmental stage and visual morphology. The above ground downward growing gynophores (with green or purple color, 5–10 cm in length) were assigned as stage 1 (S1). The stage 2 (S2) gynophores were those that buried in the soil for about three days with thicker diameter than S1 gynophores. S2 gynophores were white in color, the enlargement of the ovary region was not observed. Stage 3 (S3) gynophores were those that buried in soil for about nine days. The ovary regions of S3 gynophores were obviously enlarged. About 5 mm tip region of gynophore was manually dissected, frozen in liquid nitrogen and stored at −80 °C for the following experiments. Two biological replicates were prepared for each stage. These samples were referred as S1-R1 and S1-R2, S2-R1 and S2-R2, S3-R1 and S3-R2 throughout the manuscript.

### Small RNA and degradome library construction and sequencing

Total RNAs were extracted from peanut gynophores using CTAB reagent. For small RNA library construction, 18 to 30 nt small RNAs were fractionated through polyacrylamide gel electrophoresis and ligated with 5′ and 3′ RNA adapter by T4 RNA ligase. Reverse transcription reaction and a short PCR were performed to obtain sufficient cDNA for sequencing. To identify the potential targets, two degradome libraries were constructed from aerial grown gynophores (named as D1) and gynophores that buried into soil (named as D2) separately. In brief, poly(A) RNAs that possess a 5′-phosphate were extracted and ligated to a RNA adaptor containing a 3′ MmeI recognition site by T4 RNA ligase. Reverse transcription reaction and a short PCR were performed to obtain double stranded DNA. The DNA product was purified and digested with MmeI. Then a double stranded DNA adaptor was ligated to the double-stranded DNA. The ligated products were amplified by PCR and gel-purified for sequencing. All small RNA and degradome libraries were submitted to BGI (Shenzhen, China) for 49-bp single-end sequencing on the Illumina HiSeq 2000. The raw sequence data of small RNA library and degradome library were available at NCBI Short Read Archive (SRX2374091 and SRX1734291).

### Bioinformatics analysis of small RNA sequencing data

The raw reads were preprocessed with Fastx-toolkit pipeline (http://hannonlab.cshl.edu/fastx_toolkit/) to trim the adapter sequences and filter out low-quality sequences and repetitive reads. Reads larger than 30 nt and smaller than 18 nt were discarded. Then the clean reads were aligned to peanut reference genome (https://peanutbase.org/) using SOAP2 [51]. Only perfectly matched reads were obtained and used for subsequent analysis. Reads matched to rRNA, tRNA, snRNA, snoRNA and protein-coding genes were excluded. To identify conserved miRNAs, we aligned all reads against known miRNA registered in miRBase (Release 21.0, April 2014) allowing no mismatch. For novel miRNA identification, their corresponding precursor sequences were checked using mireap (https://sourceforge.net/projects/mireap/) to ensure the miRNA precursors have their expected secondary structures. The expression of miRNAs during peanut pod development was analyzed by reads per million (RPM). The differential expression of miRNAs was performed using DESeq package (version 2.14, http://www.bioconductor.org/packages/release/bioc/html/DESeq.html) with a criterion of |log_2_fold change| ≥ 1 and adjusted *p*-values < 0.05 [52].

### Bioinformatics analysis of degradome sequencing data

Clean reads were obtained using Fastx-toolkit pipeline (http://hannonlab.cshl.edu/fastx_toolkit/) to remove adaptor sequences and low quality reads. Only 20 and 21 nt reads that perfectly matched to peanut cDNA sequences were collected and extend to 35–36 nt by adding 15 nt of upstream sequence for potentially cleaved targets identification. The CleaveLand pipeline v3.0.1 was used to align the 35–36 nt sequence to peanut miRNAs [53]. All alignments with scores up to 5 and no mismatches at the cleavage site (between the 10th and 11th nucleotides of the miRNAs) were considered candidate targets. Tag numbers for target genes were normalized by RP10M (reads per 10 million).

### Quantitative RT-PCR analysis

The stem-loop quantitative RT-PCR (qRT-PCR) was performed to analyze the expression of miRNAs as described previously [54]. Reverse transcription reactions were performed at 16 °C for 30 min, followed by 60 cycles at 30 °C for 30 s, 42 °C for 30 s, 50 °C for 1 s and terminated by incubating at 85 °C for 5 min. *U6* was used as the internal control. For target genes, 2 μg DNase I-treated total RNA was used to synthesize cDNA using olig(dT)18 primer, and peanut actin gene was used as the internal control. Reverse transcription was performed at 42 °C for 60 min and 85 °C for 5 min. SYBR Green PCR Master Mix (Bio-Rad) was used in all qRT-PCR reactions with an initial denaturing step of 95 °C for 5 min, followed by 45 cycles of 95 °C for 5 s, 60 °C for 5 s and 72 °C for 8 s. Three biological replicates were prepared for each sample. The relative expression changes of miRNAs were calculated using the 2^-△△Ct^ method. Student’s *t*-test was used to access whether the qRT-PCR results were statistically different between two samples (*P < 0.05). Primers used in all qRT-PCR experiments were listed in Additional file 8: Table S4.

### RLM-5′ RACE

Total RNA (200 μg) from peanut gynophore was extracted using CTAB reagent and mRNA was purified using the Oligotex kit (Qiagen). RNA ligase-mediated rapid amplification of 5′ cDNA ends (RLM-5′ RACE) was performed with the RLM-RACE kit according to the manufacturer’s instructions (Clontech). The final PCR product was extracted and purified from 2% agarose gel, cloned into pMD18-T simple vector (Takara). Plasmid DNA from 10 different colonies was sequenced. Gene specific primers used for RLM-5′ RACE experiments were listed in Additional file 8: Table S4.

## Additional files

Additional file 1: Table S1. Statistics of different small RNAs from small RNA libraries. (DOCX 20 kb)
Additional file 2: Figure S1. The correlation coefficient of miRNA expression between two biological replicates of S1, S2 and S3. (TIF 7318 kb)
Additional file 3: Table S3. Detailed information of novel miRNAs in peanut. (XLS 46 kb)
Additional file 4: Figure S2. Validation of novel miRNAs by stem-loop RT-PCR. No template: no RNA was added as a negative control. (TIF 3171 kb)
Additional file 5: Table S2. The miRNA normalization, fold change and statistical significance of known or novel miRNAs in S1, S2, S3 gynophores. (XLSX 61 kb)
Additional file 6: Table S5. Statistics of different small RNAs categories by degradome sequencing. (DOCX 17 kb)
Additional file 7: Figure S3. Summary of GO classification of miRNA targets in peanut gynophore. (TIF 1258 kb)
Additional file 8: Table S4. Oligonucleotide primer sequences used for qRT-PCR. (XLS 31 kb)

## Abbreviations

3′-UTR: 3′-Untranslated Region
5′-UTR: 5′-Untranslated Region
ABA: Abscisic acid
AGO1: Argonaute1
AP2: Apetala2
ARF: Auxin response factor
BRs: Brassinolides
cDNA: Complementary DNA
CDS: Code Sequence
GA: Gibberellic acid
GO: Gene Ontology
GRF: Growth regulating factor
IAA: Indole-3-acetic acid
KEGG: Kyoto Encyclopedia of Genes and Genomes
miRNAs: microRNA
mRNAs: Messenger RNAs
NAC: NAM/ATAF/CUC2
NF-Y: Nuclear transcription factor Y
nt: Nucleotide
PPRP: Pentatricopeptide repeat protein
qRT-PCR: Quantitative Real-time PCR
RISC: RNA-induced silencing complex
RLM 5′-RACE: RNA ligase-mediated rapid amplification of 5′ cDNA ends
RP10M: Reads per 10 million clean reads
RPM: Reads per million clean reads
rRNA: Ribosomal RNA
snoRNA: Small nucleolar RNA
snRNA: Small nuclear RNA
SPL: Squamosa promoter binding like
ta-siRNAs: Trans-acting siRNAs
TCP: TEOSINTE BRANCHED1/CYCLOIDEA/PRO-LIFERATING CELL FACTOR1
tRNA: Transfer RNA
