# Supplementary material for: Small RNA profiling and degradome analysis reveal regulation of microRNA in peanut embryogenesis and early pod development
Source: BMC Genomics. 2017 Mar 2;18:220. doi: 10.1186/s12864-017-3587-8 (PMC5335773; doi:10.1186/s12864-017-3587-8)
Supplement: Additional file 1: Table S1. — Statistics of different small RNAs from small RNA libraries. (DOCX 20 kb) [file 12864_2017_3587_MOESM1_ESM.docx]

Table S1 Statistics of different small RNAs categories

| Total | S1-R1 | S1-R2 | S2-R1 | S2-R2 | S3-R1 | S3-R2 |
| --- | --- | --- | --- | --- | --- | --- |
| Clean reads | 12801776 | 13085002 | 12991630 | 13631798 | 13567428 | 13152634 |
| Mapped to genome | 10194888 | 10314602 | 10230131 | 10505281 | 11112030 | 10476270 |
| Mapped to Rfam | 298423 | 265755 | 320045 | 268446 | 204480 | 263151 |
| Mapped to repeat | 941565 | 974524 | 1008872 | 967249 | 1054796 | 967446 |
| Mapped to CDS | 754896 | 714396 | 720136 | 746968 | 772471 | 754755 |
| Mapped to miRBase | 725575 | 673341 | 666457 | 687849 | 583489 | 570536 |
| Unannotated | 6748860 | 7686586 | 7874621 | 7834769 | 8496794 | 8599662 |
| Unique |  |  |  |  |  |  |
| Clean reads | 6088689 | 6333310 | 6616605 | 6347934 | 6749227 | 6338590 |
| Mapped to genome | 4950056 | 5137499 | 5292405 | 5098563 | 5473542 | 5155109 |
| Mapped to Rfam | 30528 | 28472 | 37166 | 33831 | 28570 | 30259 |
| Mapped to repeat | 500687 | 516467 | 554777 | 505247 | 554960 | 511101 |
| Mapped to CDS | 26385 | 25432 | 27598 | 23425 | 26957 | 25144 |
| Mapped to miRBase | 20218 | 20225 | 19408 | 20094 | 20525 | 19807 |
| Unannotated | 4372238 | 4546903 | 4653456 | 4515966 | 4842530 | 4568798 |
