# Supplementary figures and images for: Small RNA profiling and degradome analysis reveal regulation of microRNA in peanut embryogenesis and early pod development
Source: BMC Genomics. 2017 Mar 2;18:220. doi: 10.1186/s12864-017-3587-8 (PMC5335773; doi:10.1186/s12864-017-3587-8)

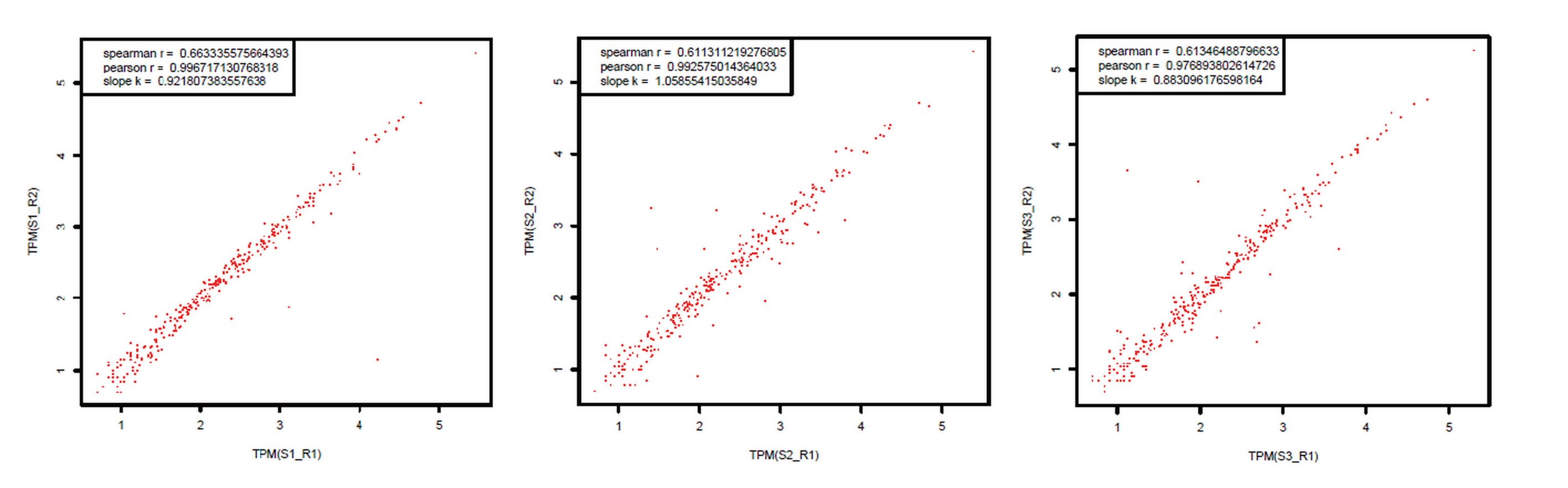

Supplement: Additional file 2: Figure S1. — The correlation coefficient of miRNA expression between two biological replicates of S1, S2 and S3. (TIF 7318 kb) [file 12864_2017_3587_MOESM2_ESM.tif]

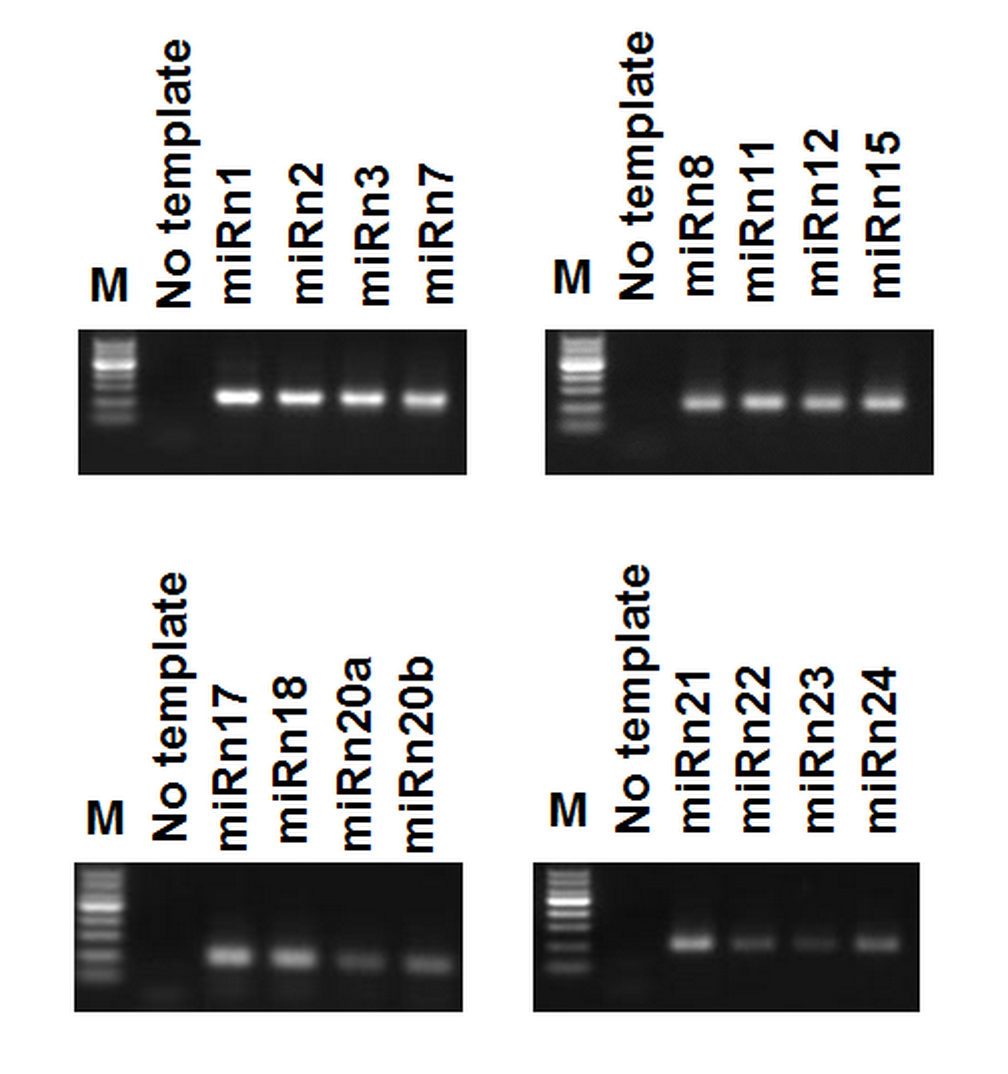

Supplement: Additional file 4: Figure S2. — Validation of novel miRNAs by stem-loop RT-PCR. No template: no RNA was added as a negative control. (TIF 3171 kb) [file 12864_2017_3587_MOESM4_ESM.tif]

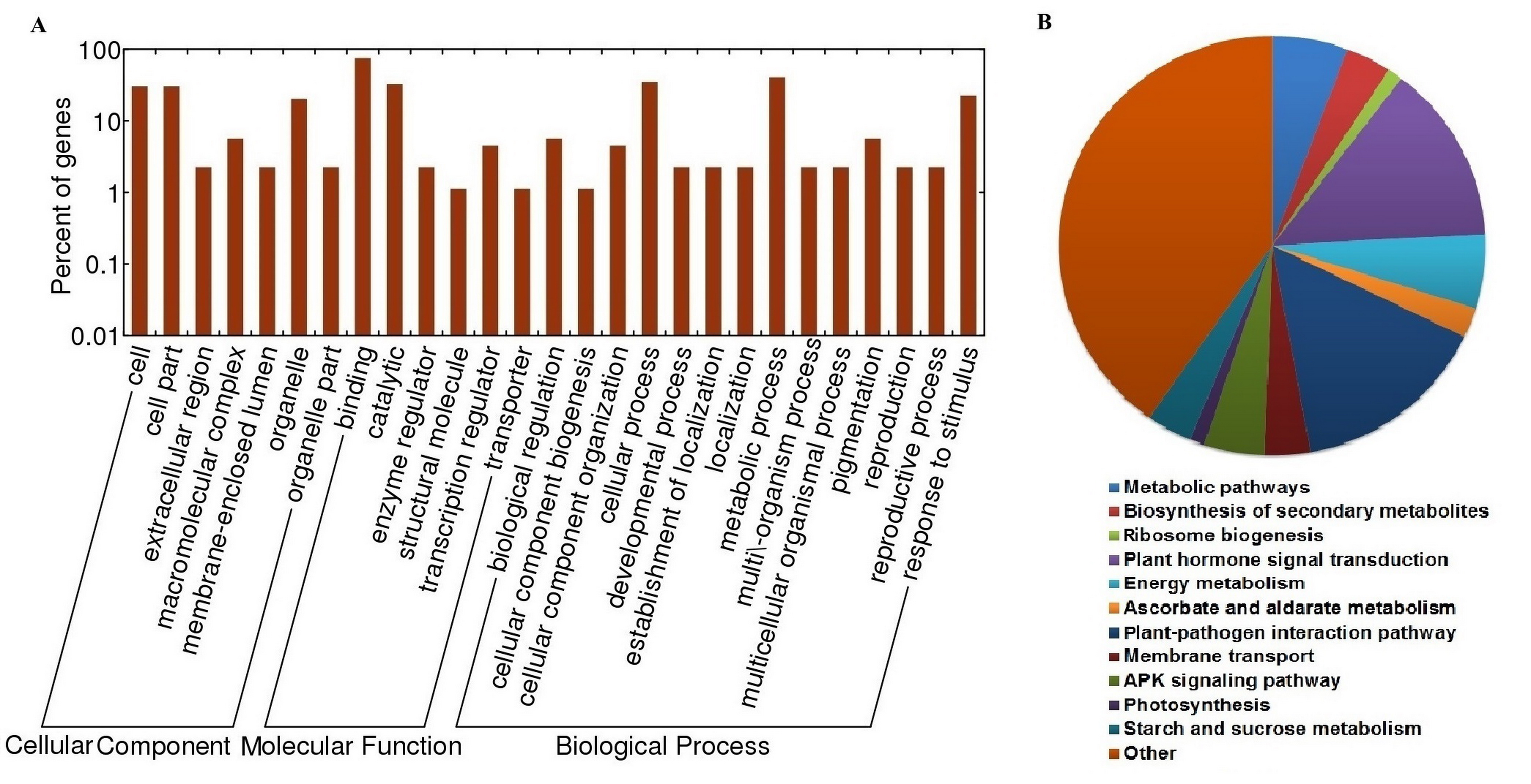

Supplement: Additional file 7: Figure S3. — Summary of GO classification of miRNA targets in peanut gynophore. (TIF 1258 kb) [file 12864_2017_3587_MOESM7_ESM.tif]
