# Supplementary material for: Small RNA profiling and degradome analysis reveal regulation of microRNA in peanut embryogenesis and early pod development
Source: BMC Genomics. 2017 Mar 2;18:220. doi: 10.1186/s12864-017-3587-8 (PMC5335773; doi:10.1186/s12864-017-3587-8)
Supplement: Additional file 6: Table S5. — Statistics of different small RNAs categories by degradome sequencing. (DOCX 17 kb) [file 12864_2017_3587_MOESM6_ESM.docx]

Table S5 Statistics of degradome reads

| Total | D1 | D2 |
| --- | --- | --- |
| Clean reads | 17205243 | 23876347 |
| Mapped to genome | 8034937 | 11841432 |
| Mapped to Rfam | 453424 | 519930 |
| Mapped to cDNA sense | 3875037 | 5890047 |
| Mapped to cDNA antisense | 3805609 | 5570135 |
| Poly N | 52483 | 15465 |
| Unannotated | 9018690 | 11880770 |
| Uninue |  |  |
| Clean reads | 7304740 | 8689034 |
| Mapped to genome | 3909547 | 4613315 |
| Mapped to Rfam | 25572 | 25838 |
| Mapped to cDNA sense | 1968898 | 2325115 |
| Mapped to cDNA antisense | 1927369 | 2275351 |
| Poly N | 22323 | 10581 |
| Unannotated | 3360578 | 4052149 |
